# Clinical recommendations for the inpatient management of lower respiratory tract infections in children and adolescents with severe neurological impairment in Germany

**DOI:** 10.1007/s00431-023-05401-6

**Published:** 2024-01-03

**Authors:** Maximilian David Mauritz, Ulrich von Both, Christian Dohna-Schwake, Christian Gille, Carola Hasan, Johannes Huebner, Markus Hufnagel, Markus Knuf, Johannes G. Liese, Hanna Renk, Henriette Rudolph, Ulf Schulze-Sturm, Arne Simon, Florian Stehling, Tobias Tenenbaum, Boris Zernikow

**Affiliations:** 1 Paediatric Palliative Care Centre, Children’s and Adolescents’ Hospital, 45711 Datteln, Germany; 2 Department of Children’s, Pain Therapy and Pediatric Palliative Care, Faculty of Health, School of Medicine , Herdecke University, 58448 WittenWitten, Germany; 3 Department of Infectious Diseases, Dr von Hauner Children’s Hospital, LMU University Hospital, 80337 Munich, Germany; 4 Department of Pediatrics I, Neonatology, Pediatric Intensive Care Medicine, and Pediatric Neurology, University Hospital Essen, 45147 Essen, Germany; 5 Department of Neonatology, Heidelberg University Children’s Hospital, 69120 Heidelberg, Germany; 6 Department of Paediatrics and Adolescent Medicine, Medical Faculty, University Medical Centre, University of Freiburg, 79106 Freiburg, Germany; 7 Department for Pediatric and Adolescent Medicine, Worms Clinic, 67550 Worms, Germany; 8 Department of Paediatrics, Division of Paediatric Infectious Diseases, University Hospital of Wuerzburg, 97080 Würzburg, Germany; 9 University Children’s Hospital Tuebingen, 72076 Tuebingen, Germany; 10 Department of Pediatrics, Goethe University Frankfurt, 60590 Frankfurt am Main, Germany; 11 University Children’s Hospital, University Medical Centre Hamburg-Eppendorf, 20246 Hamburg, Germany; 12 Pediatric Oncology and Hematology, University Hospital Homburg Saar, 66421 Homburg/Saar, Germany; 13 Department of Pediatric Pulmonology and Sleep Medicine, University Children’s Hospital Essen, 45147 Essen, Germany; 14 Clinic for Child and Adolescent Medicine, Sana Klinikum Lichtenberg, Academic Teaching Hospital, Charité-Universitätsmedizin, 10365 Berlin, Germany

**Keywords:** Severe neurological impairment, Lower respiratory tract infections, Pneumonia, Antimicrobial stewardship

## Abstract

Children and adolescents with severe neurological impairment (SNI) require specialized care due to their complex medical needs. In particular, these patients are often affected by severe and recurrent lower respiratory tract infections (LRTIs). These infections, including viral and bacterial etiology, pose a significant risk to these patients, often resulting in respiratory insufficiency and long-term impairments. Using expert consensus, we developed clinical recommendations on the management of LRTIs in children and adolescents with SNI. These recommendations emphasize comprehensive multidisciplinary care and antibiotic stewardship. Initial treatment should involve symptomatic care, including hydration, antipyretics, oxygen therapy, and respiratory support. In bacterial LRTIs, antibiotic therapy is initiated based on the severity of the infection, with aminopenicillin plus a beta-lactamase inhibitor recommended for community-acquired LRTIs and piperacillin-tazobactam for patients with chronic lung disease or tracheostomy. Ongoing management includes regular evaluations, adjustments to antibiotic therapy based on pathogen identification, and optimization of supportive care. Implementation of these recommendations aims to improve the diagnosis and treatment of LRTIs in children and adolescents with SNI.

**Table:** 

**What is Known:** *• Children and adolescents with severe neurological impairment are particularly affected by severe and recurrent lower respiratory tract infections (LRTIs).* *• The indication and choice of antibiotic therapy for bacterial LRTI is often difficult because there are no evidence-based treatment recommendations for this heterogeneous but vulnerable patient population; the frequent overuse of broad-spectrum or reserve antibiotics in this patient population increases selection pressure for multidrug-resistant pathogens.*	
**What is New:** *• The proposed recommendations provide a crucial framework for focused diagnostics and treatment of LRTIs in children and adolescents with severe neurological impairment.* *• Along with recommendations for comprehensive and multidisciplinary therapy and antibiotic stewardship, ethical and palliative care aspects are taken into account.*

**What is Known:**

*• Children and adolescents with severe neurological impairment are particularly affected by severe and recurrent lower respiratory tract infections (LRTIs).*

*• The indication and choice of antibiotic therapy for bacterial LRTI is often difficult because there are no evidence-based treatment recommendations for this heterogeneous but vulnerable patient population; the frequent overuse of broad-spectrum or reserve antibiotics in this patient population increases selection pressure for multidrug-resistant pathogens.*

**What is New:**

*• The proposed recommendations provide a crucial framework for focused diagnostics and treatment of LRTIs in children and adolescents with severe neurological impairment.*

*• Along with recommendations for comprehensive and multidisciplinary therapy and antibiotic stewardship, ethical and palliative care aspects are taken into account.*

## Introduction

Children and adolescents with severe neurological impairment (SNI) often present with complex and unique medical needs that require specialized care and attention. SNI encompasses a range of conditions characterized by significant impairments in neurological function, including motor, sensory, and cognitive abilities. These individuals often face substantial challenges in their daily lives and require comprehensive medical support to address their specific needs [1]. Other publications also describe these patients as “children with medical complexity” [2], patients with neuromuscular diseases/disorders [3, 4], or “children with severe global developmental delay” [5]. Nevertheless, the SNI definition most comprehensively describes the group of patients this treatment recommendation intends to address. Managing the medical needs of children and adolescents with SNI is a multifaceted task requiring a multidisciplinary approach. These individuals may experience difficulties in various health-related aspects, including respiratory function, mobility, nutrition, and communication. Their conditions can result in limitations of muscle control, impaired sensation, and cognitive impairments, leading to a high risk of secondary health complications. Notably, these patients are affected by recurrent, sometimes severe lower respiratory tract infections (LRTIs) [6, 7]. The causes for the increased susceptibility to LRTI are complex and multifactorial. Risk factors for LRTIs include impaired secretion clearance (lower cough stimulus, ineffective cough), restrictive lung dysfunction, development of dys- and atelectasis, and micro- and macroaspiration due to dysphagia, seizures, or gastroesophageal reflux [8–11]. Polypharmacy in this patient group can also influence bone marrow function and lead to immunological side effects, e.g., due to secondary effects of antiepileptic drugs. Some of these patients are fitted with a tracheostomy or a gastrostomy, and on rare occasions, with a suprapubic bladder catheter or jejunostomy. Due to extensive nursing care contacts, in emergency situations and due to autoinoculation, there is also an increased risk of transmitting pathogenic bacteria and viruses.

In patients with SNI, LRTIs account for a significant proportion of morbidity and are associated with an increased risk of death compared to healthy children [12–14]. In addition, increased rates of LRTI-associated complications are to be expected in patients with SNI [15]. Recurrent LRTIs can lead to progressive respiratory insufficiency and, subsequently, affecting the quality of life of these children, adolescents, and their families [16, 17]. A unique aspect of treating LRTI in these patients is the difficulty in obtaining representative microbiological samples from the lower airways [18]. In many cases, this is due to the difficulty of obtaining induced sputum from these patients. Also, contamination by colonizing pathogens of the upper respiratory tract is frequent. Furthermore, viral LRTIs often have a higher clinical severity in this patient group than in pediatric patients without SNI [19–22] and often cannot be clearly differentiated clinically from bacterial LRTIs [23].

In addition, interpretability of conventional chest radiographs may be limited, due to factors such as frequent dystelectasis, scoliosis, and chronic pulmonary changes, as well as inadequate inspiratory depth and suboptimal positioning arising from disease-related non-compliance with verbal instructions. Impaired secretion management in these patients promotes bacterial overgrowth. Many of these pathogens can form biofilms, for example, on an inserted tracheal cannula. In addition, patients with dysphagia on proton pump inhibitor therapy are at risk of pulmonary complications due to (micro-)aspiration of bacteria colonizing the stomach [24, 25].

There is an increased prevalence of multidrug-resistant (MDR) bacteria colonizing pediatric patients with SNI [26, 27]. This colonization often causes uncertainty about whether a different antibiotic therapy (ABT) is required for LRTI treatment [28, 29]. The excessive use of broad-spectrum or reserve antibiotics increases the selection pressure on MDR and is associated with acute adverse effects [30]. The following dysbiosis is more likely to pave the way for subsequent LRTIs, creating a vicious circle and constantly increasing the complexity of treatment strategies.

Patients with a tracheostomy, in particular, are frequently and chronically colonized with MDR bacteria. Colonizing MDR bacteria can cause major problems in this context. However, not all patients with tracheostomy experience frequent LRTI. This complication is more likely to affect those with significant respiratory secretion mobilization problems. Persistent airway secretions promote persistent (endobronchial) neutrophilic inflammation, ultimately leading to structural lung disease of the lower airways (e.g., bronchiectasis and chronic atelectasis). Despite the increased incidence of LRTIs, children and adolescents with SNI are not included in current international guidelines for the treatment of community-acquired pneumonia in children and adolescents, as these guidelines refer to otherwise healthy children [31–34]. Due to the lack of controlled studies, it is currently not possible to develop evidence-based treatment recommendations for LRTI in children and adolescents with SNI according to strictly scientific criteria. Therefore, the diagnostic and therapeutic approach in clinical practice is highly inconsistent. This consensus-based recommendation is intended to promote focused diagnostics and treatment of LRTI in children and adolescents with SNI. Particular emphasis is placed on antibiotic stewardship in terms of rational antibiotic therapy in this patient group that is administered for an adequate spectrum and duration.

## Materials and methods

These recommendations were prepared on behalf of the German Society for Pediatric Infectious Diseases (Deutsche Gesellschaft für Pädiatrische Infektiologie, DGPI) and developed according to the regulations of the AWMF (Arbeitsgemeinschaft der Wissenschaftlichen Medizinischen Fachgesellschaften: working group of the German Scientific Medical Societies). Professional societies were invited to participate according to the topic of the guideline and the aforementioned regulations [35]. Six professional societies agreed to participate in the development of the clinical recommendations. Each participating society nominated one individual with specialized expertise in the topic: DGKJ (German Society for Pediatrics and Adolescent Medicine) represented by U.v.B., DGPI (German Society for Pediatric Infectious Diseases) represented by T.T., DGP (German Society for Palliative Medicine) represented by B.Z., GNP (Society for Neuropediatrics) represented by M.K., GNPI (Society for Neonatology and Pediatric Intensive Care Medicine) represented by C.D.-S., and GPP (Society for Pediatric Pneumology) represented by F.S. The literature search was carried out in September 2022 as a MEDLINE Literature search with the search terms “pneumonia” or “lower respiratory tract infection” AND “neurological impairment” or “neuromuscular disease” or “neurodegenerative diseases” or “developmental disabilities” or “learning disability” or “medical complexity” or “developmental delay” AND “child*” or “pediatric*” or “infant*” or “adolescent*”. The selection of the inclusion or exclusion of the literature was made by the panel of experts. An initial draft of treatment recommendations was developed based on the literature available on the topic. Two online conferences were held in January 2023, and the recommendations were discussed until a consensus was reached. A revised draft of the recommendation was sent to the entire expert group in February 2023. After approval by the expert group, the recommendations were consented online by the whole expert group. Finally, the manuscript was submitted to the professional societies for review and approval. Subsequently, the final version was sent to all experts for final review and approval. The German version of the recommendations has been published in the AWMF guideline register [36]. All members of the expert group reported potential conflicts of interest.

## Results

### Concerned patients and scope of the recommendations

This treatment recommendation primarily covers the therapy of children with SNI admitted to hospitals as well as in inpatient long-term care facilities including residential facilities for long-term ventilator-dependent patients, or children’s hospices with specialist care. The need for inpatient hospital treatment for these patients must be considered on a very individual basis. The criteria for severe pneumonia such as severe dyspnea, dehydration, prolonged recapillarization time, cyanosis, apnea, or impaired consciousness are indicative [34]. Recommendations are made for pediatric patients with motor and/or cognitive impairments and whose medical complexity requires interdisciplinary and multimodal support in activities of daily living [1], such as patients with severe cerebral palsy, neurodegenerative diseases, encephalopathies, syndromic diseases, progressive muscular or metabolic diseases, severe traumatic brain injury, or unfavorable courses of cerebral tumor disease.

### Diagnostics

Clinical signs of LRTI in these patients may include tachy- or dyspnea, fever, cough, more frequent thick, viscous, and discolored respiratory secretions, new onset or increased oxygen demand, or new onset of pathologic auscultation findings. However, nonspecific signs, such as gastrointestinal symptoms, weight loss, increased restlessness, or decreased exercise tolerance, may also occur. Therefore, a thorough diagnostic workup is essential in this subset of patients. In patients who present as acutely unwell, timely recognition of septic shock and other sepsis-associated organ dysfunction and adequate treatment should be provided [37].

A detailed history and physical examination should be performed when patients are admitted to the hospital. In addition, vital signs such as temperature, respiratory rate, transcutaneous oxygen saturation, blood pressure, and diuresis should be obtained. The laboratory examination should include blood count, alanine aminotransferase, aspartate aminotransferase, creatinine, C-reactive protein, and blood gas analysis, including sodium, potassium, and calcium. In severe disease with clinical sepsis, a blood culture set should also be taken (aerobic and anaerobic; ideally directly during aseptic placement of the indwelling venous cannula). In children with long-term central venous access devices or catheters, a blood culture set (aerobic and anaerobic) should be obtained from the device/catheter.

For microbiological diagnostics, deep respiratory tract secretions should be obtained as far as possible and examined for pathogenic bacterial agents. The collection of secretions can ideally be done by sampling sputum or nasopharyngeal aspirate secretions after inhalation with hypertonic saline (e.g., 2–4 ml). When critically interpreting these findings, it should be noted that such samples are contaminated with bacteria originating from the upper respiratory tract or nasopharynx. Tracheal secretions should be collected from tracheostomy with a sterile suction catheter. Detecting multidrug-resistant gram-negative bacteria (MDR-GNB) in throat swabs or nasopharyngeal secretions does not automatically mean that these bacteria are causative agents of LRTI. For differential diagnosis, throat swabs should be tested (e.g., multiplex polymerase chain reaction) for respiratory viruses, such as severe acute respiratory syndrome coronavirus type 2, respiratory syncytial virus, influenza A and B, human metapneumovirus, parainfluenza virus, adenovirus, and rhinovirus. If severe mycoplasma pneumonia is strongly suspected, mycoplasma PCR should be performed before starting macrolides or doxycycline. A positive result does not prove mycoplasma LRTI, but a negative result will most likely exclude respiratory mycoplasma infection [38]. For inpatient admission, screening for MDR bacteria should be performed according to local hospital hygiene guidelines (e.g., nasopharyngeal combination swab for methicillin-resistant *Staphylococcus aureus* (MRSA), anal swab for MDR-GNB, swab of the gastrostomy or tracheostomy, and entry sites of devices, if inflamed). MDR screening is performed primarily for hospital hygiene (infection prevention) reasons. MDR bacteria colonizing the child may not necessarily be the causative agent of a LRTI; positive findings (including previous findings) must be critically evaluated [39].

For further evaluation, ultrasonography of the thorax is recommended for suspected pleural effusion or empyema. Not every patient with LRTI requires a chest X-ray (CXR). However, in cases of new onset or worsening hypoxemia (or ventilatory dysfunction) or unclear suspected diagnosis, a CXR should be considered. In patients requiring intensive care or those with recurrent LRTI or difficult-to-interpret conventional radiographic findings, computed tomography (CT) of the chest may be considered during the infection-free interval.

### Initial treatment

All patients should receive adequate symptomatic therapy with weight-adjusted hydration, antipyretic therapy for persistent fever, respiratory aid and oxygen therapy if transcutaneous saturation is less than 92%, and inhaled bronchodilator and secretolytic therapy if needed. Supportive physiotherapeutic respiratory therapy should be given if secretions are retained. In case of severe disease with respiratory insufficiency (oxygen demand, hypercapnia), the responsible pediatric pneumologists, infectious disease specialists, and intensive care specialists should be consulted at an early stage (e.g., indication for intensification of an established respiratory therapy or the start of non-invasive or invasive ventilation, high-flow respiratory aid, or cough assistant). Severe dyspnea (complaints from the patient or immediate caregiver are binding) should be treated pharmacologically, for instance, with regular or continuous administration of strong opioids. Drug administration can be either oral (or via gastric tube) or intravenous. In the case of dyspnea attacks, these can be supplemented with weight-adjusted intranasal fentanyl [40, 41].

In principle, if there is no clinical response to ABT, there should be a discussion before switching (or escalating) ABT on whether the lack of clinical response is attributable to other reasons, notably inadequate secretion management so that an improvement in symptomatic treatment can improve the patient’s situation independently of ABT.

### Suspected viral LRTI

If influenza is strongly suspected (e.g., influenza is detected in a symptomatic contact person) or influenza viruses are detected in respiratory secretions, a 5-day course of oral (or gastric tube) therapy with oseltamivir should be started as soon as possible. Still, antiviral treatment of influenza infections is under debate [42, 43]. Treatment of SARS-CoV-2 infection in this at-risk population should follow the current coronavirus disease 2019 treatment guidelines of the respective national professional societies [22]. In case of clinical deterioration, re-evaluation for bacterial coinfection should be performed after 48–72 h. In patients with, or immediately after, influenza infection, pneumococci, and *S. aureus* are the most common pathogens of bacterial coinfection [44].

### Suspected bacterial LRTI

Initial ABT should be based on a group classification. Here, patients with community-acquired LRTI or aspiration pneumonia are summarized as group I, whose primary therapy should be an aminopenicillin plus a beta-lactamase inhibitor. The most common pathogens of childhood community-acquired pneumonia (including pneumococci, *Haemophilus influenzae*, *Moraxella catarrhalis*, group A streptococci, and methicillin-susceptible *S. aureus*, with the exception of *Mycoplasma* spp.) are adequately treated with this therapy [45, 46]. This combination also covers many of the anaerobic species relevant to these patients. It also applies to pneumococci with reduced penicillin susceptibility (not penicillin-resistant isolates) at the high dose of aminopenicillins [47]. Depending on the severity of the LRTI, initial therapy may also consider known colonization of the nasopharynx or experience with previous episodes of hospitalized LRTI in cases of evident aspiration pneumonia [48]. Group II patients are those with chronic lung disease, tracheostomy, and frequent recurrent LRTI. Initial therapy with piperacillin-tazobactam is recommended. The extension to piperacillin-tazobactam primarily means an increase in efficacy in the gram-negative range against isolates with beta-lactamases that are inhibited by tazobactam and against *P. aeruginosa*. Penicillin-resistant pneumococci are not piperacillin-tazobactam-sensitive; they require therapy with ceftriaxone or vancomycin (teicoplanin), for example [49]. Specific treatment recommendations are summarized in Table 1.

**Table 1:** Empiric antibiotic therapy for suspected bacterial LRTI

**Patient group**	**Therapy recommendation**
Group I: Ambulatory-acquired LRTI or aspiration pneumonia	Ampicillin-sulbactam intravenously 150 mg/kg/day related to the ampicillin fraction every 8 h (maximum dose 2/1 g) ***or***
	Amoxicillin-clavulanic acid 7:1 orally or by gastric tube 50(-80*) mg/kg/day related to the ampicillin fraction every 8 h (maximum dose 875 mg amoxicillin) ***or***
	Sultamicillin orally or by gastric tube 50(-80*) mg/kg/day related to the ampicillin fraction every 8 h (maximum dose 750 mg sultamicillin)
Group II: Patients with chronic lung disease or patients with tracheostomy and frequent recurrent LRTI	Piperacillin-tazobactam intravenously 300 mg/kg/day related to the piperacillin fraction every 6–8 h (maximum dose 4/0.5 g)

*LRTI* lower respiratory tract infection

*The high dose of amoxicillin/ampicillin (80 mg/kg/day) is supposed to be reserved for the treatment of pneumococcal infections with reduced penicillin susceptibility; this should be taken into account depending on the local epidemiological situation

Critically ill patients should be treated primarily with piperacillin-tazobactam irrespective of group. If LRTI is present along with septic shock or sepsis with multiple organ failure, the initial therapy should be with meropenem plus vancomycin, for example.

In patients with SNI and LRTI who are colonized with MRSA and are not critically ill, the question arises whether the MRSA isolate should be considered when selecting initial empiric therapy. Including MRSA coverage is mandatory only if there is evidence of complicated LRTI (abscessing or associated with pleural empyema) or if the MRSA isolate expresses Panton-Valentine leukocidin (PVL, usually community-acquired MRSA). The reason for mandatory co-treatment in PVL positivity is the risk of necrotizing pneumonia [50]. In principle, a look at the resistogram of the respective MRSA isolate is helpful (if available) because treatment options other than vancomycin or teicoplanin may be available here (e.g., clindamycin, doxycycline, fosfomycin, and cotrimoxazole).

Critically ill patients with SNI and LRTI colonized with MRSA should be empirically treated with vancomycin or alternatively with linezolid (off-label therapy) if the resistogram is unknown. There was no clear preference in the expert group regarding this, though vancomycin requires clinical and laboratory monitoring of renal function and therapeutic drug monitoring. For patients receiving initial combination therapy, the “antibiotic timeout” is particularly important because often, after 48–72 h, the combination therapy can be switched to a targeted monotherapy.

### Ongoing management

**Fig. 1:**
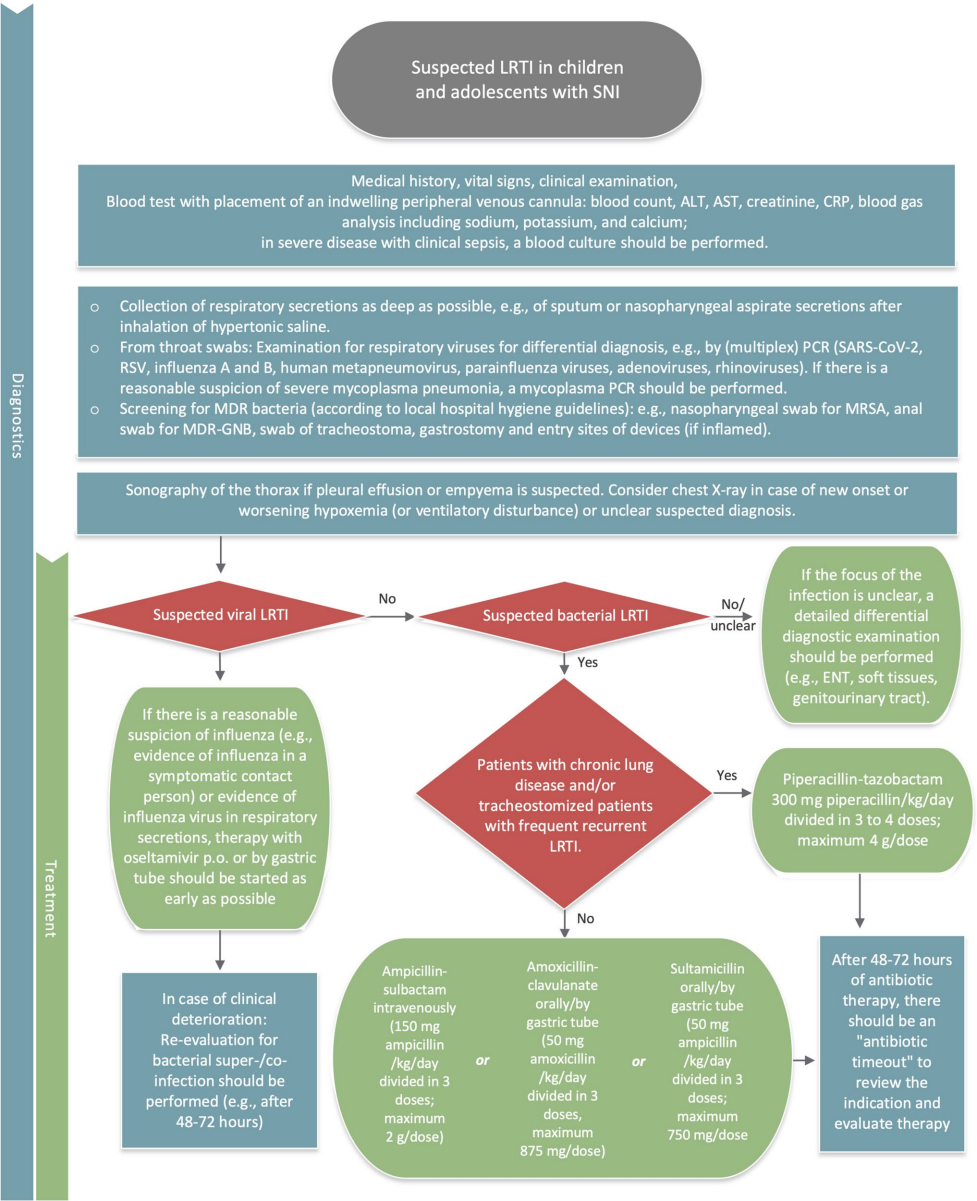
Initial diagnostic and therapeutic measures for lower respiratory tract infections in children and adolescents with severe neurologic impairment. Kg, kilogram; LRTI, lower respiratory tract infection; MRSA, methicillin-resistant *Staphylococcus aureus*; MDR, multidrug-resistant; PCR, polymerase chain reaction; RSV, respiratory syncytial virus; SARS-CoV-2, severe acute respiratory syndrome coronavirus type 2; SNI, severe neurological impairment

After 48–72 h of antibiotic therapy, an “antibiotic timeout” should be performed to review the indication and evaluate the current therapy. This timeout should already be planned when the patient is admitted, preferably with an infectious disease consultation. All available information should be collected and reviewed to evaluate the likelihood that a bacterial infection is present in the patient. The clinical course under antibiotic therapy, X-ray/CT findings, laboratory findings, and the microbiological results of bacterial and virological diagnostics should be taken into account. Initial diagnostic and therapeutic measures are summarized in Fig. 1.

### If the patient’s clinical condition improves

If bacterial infection is considered unlikely during the “antibiotic timeout,” ABT should be discontinued. If a bacterial infection is suspected and the patient’s condition improves under the initiated therapy, then the ABT should be adapted where feasible to target the specific pathogen that may have been detected (e.g., penicillin, ampicillin, or amoxicillin instead of ampicillin-sulbactam). ABT can also be changed from intravenous to oral therapy (respectively, therapy via gastral tube) if possible.

### If the patient’s clinical condition remains poor

If the patient’s condition remains persistently poor, attempts should be made to improve all aspects of supportive care (e.g., inhalation, secretolysis, physical therapy, positioning treatment, respiratory and cough support, dyspnea treatment, and pain management). If there are new relevant findings upon clinical examination or imaging, other diagnoses or complications should be considered. After infectious diseases and/or pulmonary consultation, a repeat CXR or ultrasound can determine the presence of pleural empyema. Bronchoalveolar lavage may be necessary. If pathogens are detected and resistogram results are significant, a targeted change of ABT should be performed. For LRTI due to *Pseudomonas* spp., piperacillin-tazobactam should be administered every 6 h. In addition, if vascular access permits, the infusion duration should be extended to 3–4 h per administration.

### If the patient’s clinical condition worsens significantly

If the patient’s condition worsens significantly during therapy, all aspects of supportive care (e.g., inhalation, secretolysis, physical therapy, positioning treatment, respiratory and cough support, dyspnea treatment, and pain management) should be optimized first. In the case of pathogen detection from respiratory specimens and available resistogram results, a targeted change of ABT should be performed. If no pathogens are detected, therapy should be escalated. In the case of group I patients treated with an aminopenicillin plus beta-lactamase inhibitor, therapy should be changed to piperacillin-tazobactam.

The various options for escalating therapy in patients previously treated with piperacillin-tazobactam (group II) should be adapted to the individual patient. For example, patients with severe peripheral neuropathy, pre-existing renal dysfunction, or sensorineural hearing loss should not receive aminoglycosides. The alternatives listed in Table 2 should lead to a more restrictive use of meropenem.

**Table 2:** Possible escalation of antibiotic therapy (in the absence of pathogen identification)

**Patient group**	**Therapy recommendation**
Group I: Escalation of aminopenicillin plus beta-lactamase inhibitor to	Piperacillin-tazobactam intravenously 300 mg/kg/day related to the piperacillin fraction every 6–8 h (maximum dose 4/0.5 g), subsequent re-evaluation after 48–72 h
Group II: Escalation of piperacillin-tazobactam (alphabetical)	*Combination of* piperacillin-tazobactam *plus* intravenous tobramycin* 7.5 mg/kg/day every 24 h ***or***
	*Combination of* piperacillin-tazobactam *plus* fosfomycin intravenously 200 mg/kg/day every 8 h (maximum dose 5 g) ***or***
	*Switch to* cefepime intravenously 150 mg/kg/day every 8 h, extend infusion duration to 2–4 h from second administration, maximum dose 2 g *plus* fosfomycin intravenously 200 mg/kg/day every 8 h, maximum dose 5 g ***or***
	*Switch to* ceftazidime intravenously 150 mg/kg/day every 8 h, maximum dose 2 g *plus* tobramycin* intravenously 7.5 mg/kg/day every 24 h ***or***
	*Switch to* levofloxacin intravenously or oral/by gastric tube (infusion for min 1 h), < 5 years, 20 mg/kg/day every 12 h; > 5 years, 10 mg/kg/day every 24 h; maximum dose 750 mg ***or***
	*Switch to* meropenem intravenously 60 mg/kg every 8 h, maximum dose 1 g, extend infusion duration to 2–4 h from second administration onwards

*LRTI* lower respiratory tract infection

*Potentially nephro- and ototoxic. Therapeutic drug monitoring is required to document adequate elimination

Ciprofloxacin is the preferred fluoroquinolone for infections caused by *P. aeruginosa* [51]. In principle, restraint is required in the use of fluoroquinolones in children due to the fear of long-term selection of MRD bacteria and possibly also due to possible neurotoxicity. Including fluoroquinolones in this list must not be misinterpreted as a “license for uncritical use,” especially in children and adolescents with SNI who are treated as outpatients.

### Duration of antimicrobial therapy

Antibiotic therapy, with clinical efficacy usually seen after 48–72 h, should be given for 5–7 days in patients with SNI. A longer duration may be required in complicated LRTI (e.g., parapneumonic effusion and pleural empyema) or depending on clinical response. For bronchiectasis, a duration of 10–14 days is recommended. Sequential oral therapy is possible according to the results of the resistogram.

### Prevention of (recurrent) LRTIs and considerations for future treatments

In patients with SNI and recurrent bacterial LRTIs caused by gram-negative infectious agents, inhalation therapy with agents such as tobramycin or colistin can be considered (depending on the antibiogram) for prophylaxis or pathogen eradication, similar to what is employed for cystic fibrosis (CF) patients (e.g., 28 days on/off cycles) [52]. In patients with the aforementioned conditions, to prevent invasive or pulmonary infections, vaccinations should be administered according to the current national recommendations. Vaccination against the most common bacterial LRTI pathogens can also counteract the overuse of antibiotics and subsequent antibiotic resistance [53]. For pneumococcal immunization, we recommend sequential vaccination initiated with 13-valent or higher conjugate vaccine and completed with 23-valent polysaccharide vaccine after 6–12 months [54]. In addition, seasonal immunization should be given to protect against influenza.

Analogous to the procedure for CF patients, a strategy for future empiric ABT should be defined for children and adolescents with SNI and recurrent bacterial LRTI. This plan should be accessible to all medical personnel involved in their care, such as including it in the patient’s file note or as a recommendation in their hospital discharge letter. For patients with recurrent bacterial LRTIs for whom oral medications have not been effective in previous LRTIs, early implantation of a long-term central venous access device can help both the patients and the medical staff involved.

*Ethical and palliative care aspects.*

The medical treatment of children and adolescents with severe neurological disorders, regardless of acute LRTI, has the overarching goal of maximizing quality of life until the end of life. Children and adolescents with SNI often have a limited life expectancy and are burdened by various symptoms with limited causal therapeutic treatment options. Depending on the complexity of disease, specialized palliative medical co-treatment should be considered.

Before implementing any therapeutic measure, both a medical indication and the (presumed) will of the patient are required. In situations involving life-threatening crises and infections in the context of the SNI-related illnesses described above, palliative and symptom-relieving therapy may take precedence during the treatment over curative goals focused on healing and prolonging life. Individual therapy goals and decisions should be made through specialist consultation involving participatory decision-making, either with patients capable of giving consent or with their legal guardians or caregivers. The local clinical ethics committee, palliative physicians, hospital chaplains, or psychologists can provide support during this process. Regardless of the goal of the therapy, the aim is always to alleviate distressing symptoms such as pain, shortness of breath, anxiety, or restlessness and to provide holistic, family-centered support.

In patients with life-limiting diseases, there may be instances where certain burdensome, life-prolonging intervention measures are no longer desirable, with a greater emphasis placed on specialized palliative care. Therapeutic limitations should be considered when clinical experts determine that implementing new interventions such as intensive therapy escalation (e.g., ventilation and central catheter placement) are highly likely to only prolong the dying phase or otherwise excessively burden the patient rather than overcome acute medical deterioration.

Advance care planning (ACP) at times when patients are not in a critical phase of life helps glean the wishes of patients and their guardians for the different phases of disease deterioration. ACP should be conducted for all patients with SNI [55]. Part of this process can be an “advance declaration on emergency situations,” which predefines specific treatment preferences to be communicated by the aforementioned caregivers or patients capable of giving consent in the event of deterioration in the underlying disease or associated complications (e.g., severe or recurrent LRTI). The existence of such an advance directive does not absolve the treatment provider of their obligation to coordinate the current treatment as early as possible with representatives, and if necessary, with patients capable of giving consent. The expression of the patient’s will by patients capable of giving consent or by the patient’s legal guardians or caregivers always takes precedence over any advance directive.

## Summary of recommendations

Managing the medical needs of children and adolescents with SNI is a multifaceted task that requires a comprehensive multidisciplinary approach. The aforementioned recommendations aim to provide age-specific guidance for optimizing the diagnosis and treatment of lower respiratory tract infections in children and adolescents with SNI. All children and adolescents with SNI and acute LRTI should receive adequate symptomatic therapy, including inhalation, secretolysis, physiotherapy, positioning, respiratory and cough support, dyspnea treatment, and pain therapy as needed. They should receive adequate medical and microbiological diagnostics in line with these recommendations, including a workup concerning possible differential diagnoses. When starting ABT, an “antibiotic timeout” after 48–72 h should be established to check the indication and evaluate the therapy in a multidisciplinary team of clinicians and infectious disease specialists or an antibiotic stewardship team. In the case of suspected bacterial LRTI, a calculated antibiotic therapy with an aminopenicillin plus a beta-lactamase inhibitor should be given, and in patients with known chronic lung disease and patients with tracheostomy and frequently recurring LRTI, piperacillin/tazobactam is considered the drug of choice. During the course of therapy re-evaluation during the “antibiotic timeout,” the indication for an initially scheduled ABT or initially withheld ABT should be re-evaluated again within repeated “antibiotic timeouts.” The detection of a respiratory virus infection may help in the decision to stop an initiated ABT. However, clinical and laboratory findings should also be considered. Detecting a respiratory virus does not exclude the possibility of a bacterial (co-)infection [56, 57]. If a bacterial LRTI is likely, ABT adjustment for relevant microbial findings from the respiratory specimen should be made at this time. This should involve focusing ABT and can involve an intravenous-to-oral switch. In the absence of improvement of the clinical condition, all elements of symptomatic therapy should first be optimized. In the case of significant deterioration, empirical escalation of therapy should be performed in the absence of pathogen evidence from respiratory material or blood cultures.

## Discussion

The rationale for the ABT proposed in these recommendations arises in particular from the transfer of experience in the treatment of patients with CF. Similar to this patient group, in pediatric patients with SNI, pathogens such as *Pseudomonas aeruginosa*, *Staphylococcus aureus*, and Enterobacteriaceae play an important role in the development of LRTIs in addition to the typical pathogens of community-acquired pneumonia (CAP) in otherwise healthy children and adolescents [58–61]. Nevertheless, the more intensive (broader-acting and longer-lasting) ABTs in cystic fibrosis patients should not be applied blindly to all patients with SNI and LRTI due to the lack of controlled studies and the risk of long-term selection of MRD bacteria in the sense of a “one size fits all” approach. The proposed empiric therapy options cover common bacterial pathogens of CAP in children [46], *S. aureus*, and many GNB [62, 63] as well as rare cases of anaerobic LRTIs [64] at the same time. If these therapies fail and the pathogen is undetected, we assume that the most likely cause is an insufficiently treated gram-negative pathogen. These considerations lead to the rationale for expanding the spectrum of activity by either switching from aminopenicillin plus beta-lactamase inhibitor to the broader gram-negative active piperacillin-tazobactam or further expanding the gram-negative spectrum of the existing piperacillin-tazobactam therapy. In this context, there is favorable evidence from CF therapy for the combination therapy of a beta-lactam antibiotic or a *Pseudomonas*-active cephalosporin [65, 66] with tobramycin [67, 68] or fosfomycin [69–71]. Extended infusion of beta-lactam antibiotics has been shown to be effective and, depending on the clinical situation, is an option to increase the efficacy of ABT [72]. With levofloxacin, we propose a second-line oral treatment option for patients for whom intravenous administration is not possible or not desired due to the individual situation. Levofloxacin has good bioavailability with a broad spectrum of activity against most gram-negative and gram-positive bacteria [51]. All options are intended to offer an alternative treatment choice alongside the use of carbapenems for non-critically ill patients. Inhaled ABT, especially with tobramycin, can support acute treatment [73–75].

In addition to the above-mentioned established immunizations against common bacterial pathogens such as *Haemophilus influenzae* and *Streptococcus pneumoniae*, as well as seasonal immunization against influenza, there will be further options for vaccination or immunization against RSV in the future [76]. These should be applied within the framework of the respective national approval and recommendations in order to prevent severe courses of LRTI caused by RSV.

Implementation of these recommendations may improve the diagnostics and directed therapy of LRTI in these patients. In particular, the targeted use of antibiotics should prevent the development of resistance in colonizing bacteria.

### Limitations and critical research questions

Few studies exist on evidence-based prevention and treatment of lower respiratory tract infections in children and adolescents with severe neurological impairment. The recommendations listed here are consensus-based expert recommendations. Research questions for future evidence-based recommendations include the following: Which pathogens cause LRTI in children and adolescents with SNI? Which interventions have a preventive effect on developing lower respiratory tract infections? What prophylactic therapies protect against recurrent lower respiratory tract infections (including consideration of resistance development)? What calculated antibiotic therapy and escalation strategy for children and adolescents with severe neurological impairment has a reasonable response rate without increased risk of resistance development of colonizing bacteria? What is the optimal length of therapy for LRTI in children and adolescents with SNI?

## Abbreviations

ABT: Antibiotic therapy
ACP: Advance care planning
CAP: Community-acquired pneumonia
CF: Cystic fibrosis
CXR: Chest X-ray
GNB: Gram-negative bacteria
kg: Kilogram
LRTI: Lower respiratory tract infection
MRSA: Methicillin-resistant *Staphylococcus aureus*
MDR: Multidrug-resistant
PCR: Polymerase chain reaction
PVL: Panton-Valentine leukocidin
RSV: Respiratory syncytial virus
SARS-CoV-2: Severe acute respiratory syndrome coronavirus type 2
SNI: Severe neurological impairment
